# Emergence and Control of Zoonotic Viral Encephalitides

**DOI:** 10.3201/eid1012.041026

**Published:** 2004-12

**Authors:** Marguerite Pappaioanou

**Affiliations:** * Centers for Disease Control and Prevention, Atlanta, Georgia, USA

The viral encephalitides of Eastern, Western, and Venezuelan equine encephalitis viruses have been of public health concern for years. Over the last decade, several outbreaks caused by emerging zoonotic viral encephalitides, such as West Nile virus in North America and Nipah virus in Malaysia and Singapore in 1999, resulted in serious illnesses and deaths in persons, domesticated food animals, and wildlife. The Institute of Medicine has cited a number of factors that have led to these and other emerging disease outbreaks: 1) a growing human population that is moving into habitats of wildlife and domesticated livestock and poultry; 2) global climate changes that have caused changes in arthropod vector and rodent reservoir populations; 3) rapid travel and movement of people and animals worldwide; and 4) changing human behaviors (*1*). Emergence and Control of Zoonotic Viral Encephalitides is a timely book that gives an overview of agent, host, environmental, and other factors that have led to the emergence and transmission of several zoonotic viral encephalitides, including flaviviruses, alphaviruses, and rabies virus. The book also details important avenues for their control.

This book is a special issue of the Archives of Virology, and its 244 pages comprise 21 presentations that were made at a symposium on "Emergence and Control of Zoonotic Viral Encephalitides." The symposium was held April 6–8, 2003, in Les Pensieres, Veyrier du Lac, France. The first presentation gives an overview of the emergence of zoonotic viruses maintained by wildlife reservoir hosts and describes a conceptual model of processes that would account for the transmission of viruses among species. The second presentation describes the role of disease surveillance in polio eradication and the identification of emerging viral encephalitides. The third presentation gives an overview of the mechanisms of genetic changes and neurovirulence of encephalitogenic arboviruses.

The following 13 presentations include overviews of molecular determinants of virulence of West Nile virus in North America, genetic determinants of Venezuelan equine encephalitis virus, evolution and dispersal of encephalitic flaviviruses, and West Nile and other zoonotic viruses in Russia. Presentations that follow address lyssaviruses and henipaviruses transmitted by frugivorous bats, host-management strategies of novel viral encephalitides associated with bats, regulation of transcription and the nature of the cell receptor with regard to henipaviruses, and entry machinery of flaviviruses. Also included are presentations on persistent infection and suppression of host response by alphaviruses, subversive neuroinvasive strategy of rabies virus, neurovirulence and host factors in flavivirus encephalitis, regulation of apoptosis by viruses infecting insects, and Semliki Forest virus infection of laboratory mice as models to study the pathogenesis of viral encephalitis.

The book finishes with presentations on a novel principle of attenuation for developing new generation live flavivirus vaccines, on tick-borne encephalitis, and on a recombinant vaccine developed from a canarypox virus carrying the prM/E genes of West Nile virus that will protect horses against a West Nile virus–mosquito challenge, and on diagnosis of zoonotic viral encephalitis.

The book will be worthwhile to virologists and other infectious disease researchers and practitioners interested in the biology, virulence, and genetic evolution of viral encephalitides, and the factors involved in their emergence.

## References

[1] Institute of Medicine. Microbial threats to health: emergence, detection and response. Washington: National Academy Press; 2003. 25057653

